# The Diagnosis of Quadricuspid Aortic Valve-Induced Cardiomyopathy: Return to the Basics When in Doubt

**DOI:** 10.7759/cureus.77783

**Published:** 2025-01-21

**Authors:** Pedro J Diaz Delgado, Juan C Batlle, Juan C Ulloa Rodriguez, Jason S Phillips

**Affiliations:** 1 Internal Medicine, University of Texas Health Science Center at San Antonio, San Antonio, USA; 2 Internal Medicine, Yale New Haven Hospital, New Haven, USA; 3 Cardiology, University of Texas Health Science Center at San Antonio, San Antonio, USA

**Keywords:** aortic regurgitation, cardiac magnetic resonance, ejection fraction, left ventricle, quadricuspid aortic valve, transesophageal echocardiography, transthoracic echocardiography

## Abstract

We present a case of a 43-year-old female with a rare congenital quadricuspid aortic valve (QAV) leading to severe aortic regurgitation (AR) and acute decompensated systolic heart failure. This case highlights the diagnostic challenges associated with QAV, particularly when using transthoracic echocardiography (TTE), and emphasizes the importance of advanced imaging techniques like transesophageal echocardiography (TEE) and cardiac magnetic resonance (CMR) imaging in confirming the diagnosis. Prompt diagnosis and surgical intervention are crucial in preventing the progression of heart failure and improving outcomes.

## Introduction

Quadricuspid aortic valve (QAV) is a congenital anomaly in approximately 0.013% of the population [1]. This is considered rare, given that the most common congenital valve abnormality, bicuspid aortic valve, has an incidence of 1-2%. QAV was first described in 1847, fewer than 300 cases have been reported, and there is a higher incidence in men (62%) [2]. The most common complications are aortic regurgitation (AR) and aortic stenosis. QAV is classified according to two systems as follows: Hurwitz & Roberts (types A-H) and Nakamura (types I-IV), which categorize the valve according to the relative sizes of the valve cusps and the location of the extra cusp, respectively. Type B and type II QAV are the most common types. Management options for QAV include aortic valve replacement (AVR) or tricuspidization, with AVR preferred for advanced cases. We present a case of a 43-year-old female with a congenital quadricuspid aortic valve leading to severe AR and acute decompensated systolic heart failure.

## Case presentation

A 43-year-old female with a history of hypertension, type 2 diabetes mellitus, and suspected hemochromatosis presented with progressively worsening symptoms over the past year, including lower extremity edema, dyspnea on exertion, orthopnea, and dry cough. Her symptoms have significantly worsened over the past two weeks without any obvious precipitating factors. Initial evaluation revealed acute decompensated systolic heart failure with an estimated ejection fraction (EF) of 30-35%, a severely dilated left ventricle (LV) with global hypokinesis, and mild aortic regurgitation (AR) on transthoracic echocardiography (TTE). The patient denied a history of drug use or a family history of heart failure. Coronary angiography was negative for coronary artery disease. Given her history of hemochromatosis, a cardiac MRI was performed, revealing no myocardial iron deposition but confirming a dilated LV and moderate AR. Despite extensive work-up, no clear etiology was identified, and she was discharged with plans for follow-up.

**Figure 1:**
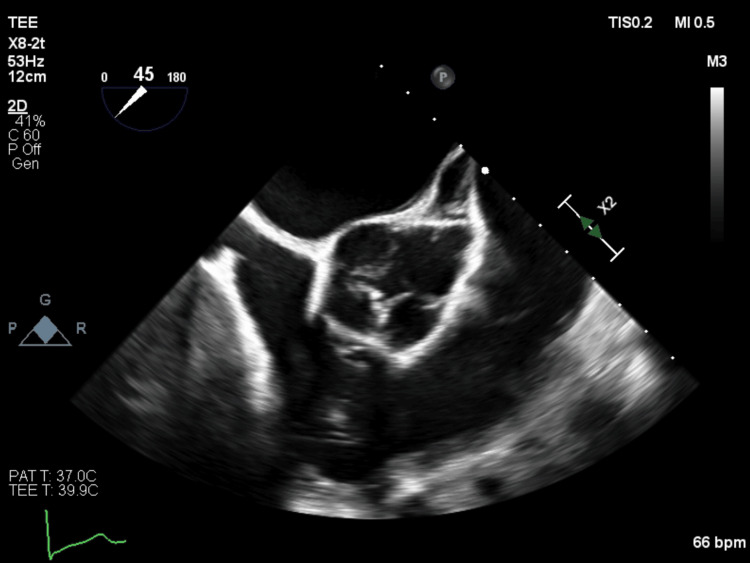
Quadricuspid aortic valve evidenced in short axis view on transesophageal echocardiogram (TEE).

**Figure 2:**
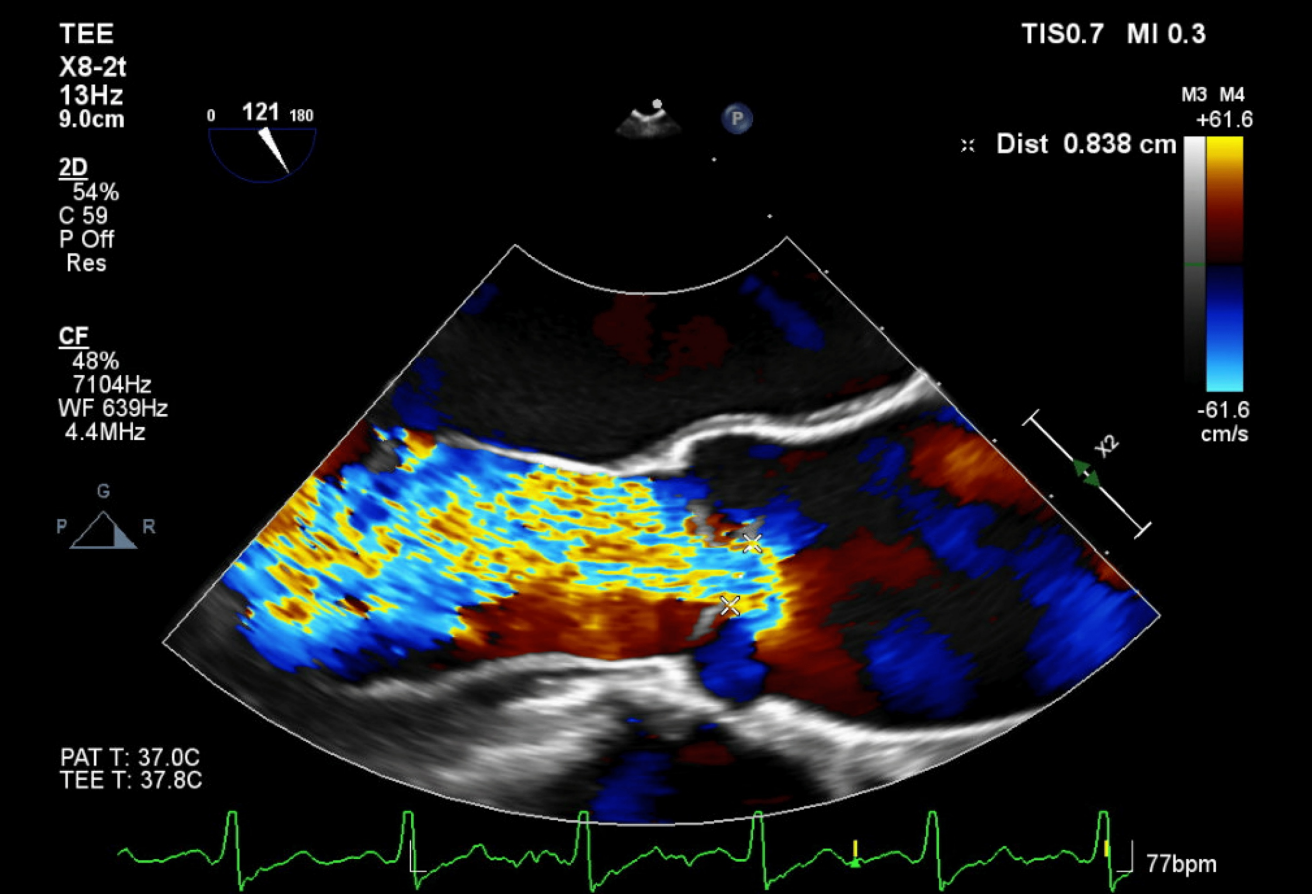
Transesophageal echocardiogram (TEE) demonstrating severe aortic regurgitation with a vena contracta of 0.83 cm.

At outpatient follow-up, her blood pressure was recorded as 116/51 mmHg, with a wide pulse pressure, which supports the possibility of severe AR in the differential diagnosis. Also, a 2/4 holodiastolic murmur was noted along the left sternal border, prompting further evaluation. A transesophageal echocardiogram (TEE) was performed, which revealed a quadricuspid aortic valve, a large AR jet, and a vena contracta of 0.8 cm which met the criteria for severe AR (Figure 1). The transesophageal echocardiogram (TEE) also showed a moderately dilated ascending aorta, measuring 4.4 cm, reflecting chronic exposure to severe AR (Figure 2).

**Figure 3:**
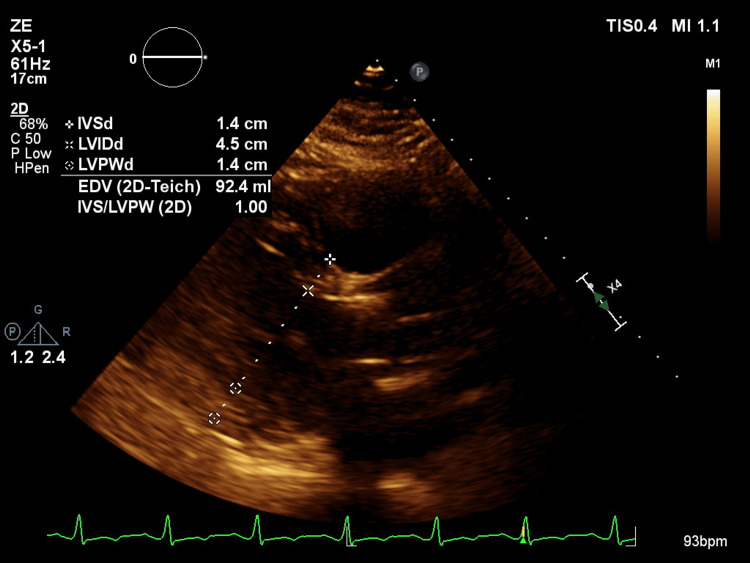
Two-month follow-up post-surgical TTE reflecting improvement of dilated cardiomyopathy and of left ventricular internal diameter during diastole. TTE: transthoracic echocardiography

Given the severity of AR and the patient's symptoms, she was referred to cardiothoracic surgery. Her Society of Thoracic Surgeons (STS) score was 0.079%, indicating low operative risk, there, she was deemed an appropriate surgical candidate. She underwent successful AVR with a #25 Hancock porcine bioprosthetic valve and ascending aorta aneurysmorrhaphy. Follow-up TTE two months later showed normalized LV size, improved LVEF to 50%, and trace AR (Figure 3). The ascending aorta remained stable at 4.3 cm; thus, moving forward, it required yearly follow-up with TTE.

## Discussion

QAV is a rare anomaly, typically found as an isolated congenital defect. However, 18-32% of cases may have associated congenital heart defects, such as atrial or ventricular septal defects, patent ductus arteriosus, or coronary artery anomalies [3]. This is relevant because, if present, patients may require additional therapies, thereby being placed at higher risk, aside from the fact that a certain intervention may be chosen in place of another due to the presence of an additional congenital cardiac defect. In the present case, the patient had no other structural abnormalities. QAV can lead to aortic regurgitation (AR) and progressive valve dysfunction. An additional cusp causes structural imbalance, leading to mechanical stress, poor coaptation, fibrosis, degeneration, and calcification, which may worsen AR and lead to aortic stenosis. As AR worsens, LV dilation and hypertrophy occur as compensatory mechanisms. If left untreated, QAV-induced AR ultimately can lead to heart failure and may cause aortic dilation, aneurysms, or dissection.

Aortic valve abnormalities causing severe AR can eventually lead to left ventricular (LV) remodeling and dilated cardiomyopathy. In the absence of surgical intervention, QAV with AR typically progresses from compensatory to decompensated LV remodeling due to the circulatory overload of volume and increased stress present in the LV provoking an initial increment in wall thickness; however, in time followed by ventricular dilation, resulting in reduced cardiac output and symptomatic heart failure. To avoid further morbidity and mortality, patients with severe AR are indicated to undergo intervention in multiple scenarios, namely with the presence of symptoms, if there is the development of a reduced EF or the LV dimension exceeds 50 mm. This report underscores the need for early intervention to prevent further cardiovascular complications, such as worsening heart failure, aortic dilation, and potential dissection.

While TTE remains the most widely used modality for evaluating AR, it can be limited by patient factors and operator skill. In the present case, the initial TTE reported only mild AR, which did not correlate with the patient's clinical presentation. Mild AR was reported due to poorly acquired images, again underscoring the vital role operator expertise plays in these clinical scenarios. The presence of a holodiastolic murmur during physical examination and a dilated LV prompted further work-up, including TEE. TEE provided better visualization of the valve and confirmed severe AR. The importance of using additional imaging when initial findings are inconclusive is emphasized, as relying solely on TTE reports can delay diagnosis.

Cardiac MRI is a valuable tool for assessing AR and its severity, although its limitations should be considered. In the present case, cardiac magnetic resonance (CMR) imaging was initially performed without cine imaging of the aortic valve, resulting in suboptimal evaluation. Although CMR can provide superior imaging in cases of AR compared to TTE, it requires appropriate protocol selection and operator expertise. Had cine imaging been included, the QAV may have been detected earlier. The inclusion of cine imaging would have aided in the detection of severe AR given that cardiac MRI is the gold standard for the evaluation of AR and provides high spatial resolution for the visualization of the aortic valve from multiple angles in a comprehensive manner.

In this report, a middle-aged woman with severe AR but no history of coronary artery disease or substance abuse led us to consider rare pathologies like QAV. Other differential diagnoses that were taken into account, were peripartum cardiomyopathy, restrictive cardiomyopathy, and acute pericarditis. The patient's QAV was identified as a type E valve according to the Hurwitz & Roberts classification, with one larger cusp and three others of equal size [4]. This specific morphology is characterized by a more balanced distribution of mechanical stress across the valve cusps reducing the likelihood of cusp prolapse and subsequent AR in comparison to type B QAV which has two larger and two smaller equally sized cusps that are more prone to the progression of valve incompetence. While type E QAV is less commonly associated with AR, this case highlights the need for a thorough differential diagnosis. Aortic valve replacement with a bioprosthetic valve was elected over other alternative therapies because it is the gold standard and was the most durable option in a young patient such as in the present case.

## Conclusions

This report emphasizes the importance of considering rare pathologies when evaluating aortic regurgitation and heart failure. It also highlights the limitations of common imaging modalities and the critical role of advanced imaging techniques like TEE and cardiac MRI in confirming the diagnosis and guiding treatment. Prompt recognition and surgical intervention significantly improved the patient’s condition, and yearly follow-up with TTE will be performed to monitor for any recurrence of AR, further aortic dilation, or depressed left ventricular ejection fraction.
